# Preparation and Hydrophobicity of Bionic Structures Based on Composite Infiltration Model

**DOI:** 10.3390/ma15124202

**Published:** 2022-06-13

**Authors:** Zhihong Jiang, Minghui Shen, Jiangtao Che, Hui Li

**Affiliations:** 1 School of Mechatronic Engineering, Beijing Institute of Technology, Beijing 100081, China; jiangzhihong@bit.edu.cn (Z.J.); 3120195103@bit.edu.cn (M.S.); 2 Beijing Advanced Innovation Center for Intelligent Robots and Systems, Beijing 100081, China; 3 Key Laboratory of Biomimetic Robots and Systems of Chinese Ministry of Education, Beijing 100081, China

**Keywords:** hydrophobicity, composite model, bionic structure, ultraprecision machining

## Abstract

The wettability, surface energy, structure, and morphology of a material’s surface will affect the interaction process between the material and the organism. Moreover, these factors are not independent of each other, but will affect each other, which together determine the biological surface of the material. Although two classic theories of surface wettability control have been established, including the Wenzel model and the Cassie–Baxter model, the mechanism of the microstructure parameters on the surface wettability has not been considered. This paper established a two-dimensional mathematical model of the composite wetting pattern based on microstructure parameters, revealed the mechanism of the microstructure parameters on the surface wettability, and then used ultra-precision cutting and molding composite preparation methods to quickly and efficiently prepare bionic structures, and the hydrophobic character of the microstructure was characterized by the contact angle meter, which provides theoretical support and preparation technology for the modification of the hydrophobic character of the material.

## 1. Introduction

Due to hundreds of millions of years of natural selection and biological evolution, many animal and plant surfaces exhibit different excellent functional properties, such as self-cleaning [1], drag reduction and wear resistance [2], corrosion resistance [3], and low adhesion [4]. As people continue to research and discover, these peculiar functions have a huge connection with biological surface structure and microscopic morphology. The surface layer of the lotus leaf exhibits excellent self-cleaning properties, that is, the phenomenon of “lotus leaf effect” [5]. This is because there are a large number of micron papillae on the surface layer of the lotus leaf, and there are nano-villi structures and low-energy waxy layers on the papillary structure. The effect leads to the super-hydrophobicity of the lotus leaf; the surface of shark skin [6] has a special groove structure, which can make it swim quickly in the water and achieve excellent drag reduction function. Imitating the special wettability surface in nature, researchers designed and prepared a variety of special wettability surfaces, among which the superhydrophobic surface attracted the most attention of researchers [7,8]. The super-hydrophobic surface has super waterproof and self-cleaning properties, and has important application value in industrial production and daily life. However, the superhydrophobic surface also has some defects and shortcomings [9,10]; for example, it only exhibits superphobic characteristics for water with high surface tension, and is easily contaminated by organic oil stains with low surface tension. The “Cassie” of water on superhydrophobic surfaces, the contact state, is unstable, and external forces such as high pressure and impact can easily cause the “Cassie” contact state to change to the “Wenzel” contact state and lose its waterproof properties. Therefore, the study of superhydrophobic models based on the surface microstructure becomes particularly important.

In recent years, scholars all over the world have conducted a large number of experiments and studies on superhydrophobic functional surfaces. There are many preparation methods, including sol-gel method [11], template method [12], chemical vapor deposition [13], laser-processing method [14] and so on. Many micro-nano manufacturing methods have been applied to prepare super-slip surfaces [15,16,17,18], but most of these methods are to build an additional layer of heterogeneous porous structure on the substrate material, and then refill lubricating oil to further obtain super-slip properties. Therefore, the prepared super-smooth surface substrate material and the liquid-infused porous structure layer have different physical, chemical, thermodynamic, and mechanical properties. When heated, bent, impacted, or other external forces are applied, it is easy to cause damage or damage to the super-slip surface layer.

This paper established a microstructure-based superhydrophobic model to study the mechanism of the influence of microstructure parameters on the superhydrophobicity of the material, and then prepared a microstructure with superhydrophobic characteristics through a composite processing method of ultra-precision cutting and molding. The microstructure was verified by experiments. The superhydrophobicity of the structure has important reference value for the further development and application of superhydrophobic materials.

## 2. Materials and Methods

**Figure 1:**
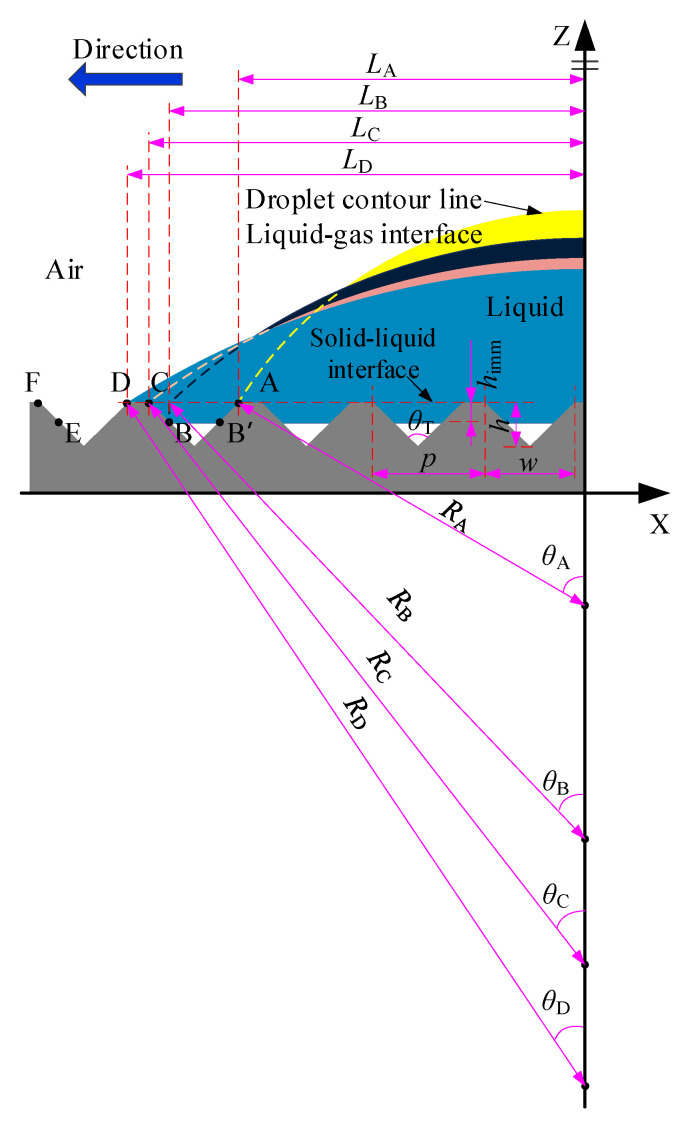
Two-dimensional model of composite infiltration mode.

Figure 1 shows the two-dimensional model in the compound infiltration mode. In this immersion mode, the liquid is not completely immersed inside the microstructure, leaving some gas inside the microstructure.

Considering the scale of the microstructure, the liquid surface of the droplet inside the microstructure can be simplified to a straight line. In the modeling, it is assumed that the depth of the liquid immersed into the microstructure is the same, and the immersion depth *h*_imm_ is introduced. Therefore, the droplet in the composite wetting mode starts from the three-phase contact point A and expands along the *X*-axis direction, first descending along the side wall of the microstructure to a point with a depth of *h*_imm_ (point B’), and crosses to the point B located on the other side wall of the microstructure, and then return to the top of the microstructure along the sidewall (point C), and continues to expand along the plane to point D, thus starting the next cycle of expansion, until the infiltration system reaches a stable state.

Considering the four positions of A, B, C, and D, we set the apparent contact angle corresponding to each contact point as *θ*_A_, *θ*_B_, *θ*_C_, and *θ*_D_, and the corresponding contour area as *A*_A_, *A*_B_, *A*_C_, and *A*_D_, respectively. At point B, the droplet profile above the apparent solid–liquid contact surface is regarded as a spherical shape, and the droplet profile below the apparent solid–liquid contact surface was simplified as a straight line. According to the geometric relationship in Figure 1, the droplet area at each stage is as follows:

(1) $$\{\begin{array}{l} A_{A}=\frac{\theta_{A}L_{A}^{2}}{\sin^{2}\theta_{A}}-\frac{L_{A}^{2}}{\tan\theta_{A}}+A_{\mathrm{inter}.}\\ A_{B}=\frac{\theta_{B}L_{B}^{2}}{\sin^{2}\theta_{B}}-\frac{L_{B}^{2}}{\tan\theta_{B}}+A_{\mathrm{inter}.}+4hh_{\mathrm{imm}}\tan\frac{\theta_{T}}{2}-3h_{\mathrm{imm}}^{2}\tan\frac{\theta_{T}}{2}\\ A_{C}=\frac{\theta_{C}L_{C}^{2}}{\sin^{2}\theta_{C}}-\frac{L_{C}^{2}}{\tan\theta_{C}}+A_{\mathrm{inter}.}+4hh_{\mathrm{imm}}\tan\frac{\theta_{T}}{2}-2h_{\mathrm{imm}}^{2}\tan\frac{\theta_{T}}{2}\\ A_{D}=\frac{\theta_{D}L_{D}^{2}}{\sin^{2}\theta_{D}}-\frac{L_{D}^{2}}{\tan\theta_{D}}+A_{\mathrm{inter}.}+4hh_{\mathrm{imm}}\tan\frac{\theta_{T}}{2}-2h_{\mathrm{imm}}^{2}\tan\frac{\theta_{T}}{2} \end{array}$$

When the droplet expands from point A to point B, we obtain:

(2) $$\{\begin{array}{l} \frac{\theta_{B}L_{B}^{2}}{\sin^{2}\theta_{B}}-\frac{L_{B}^{2}}{\tan\theta_{B}}+4hh_{\mathrm{imm}}\tan\frac{\theta_{T}}{2}-3h_{\mathrm{imm}}^{2}\tan\frac{\theta_{T}}{2}=\frac{\theta_{A}L_{A}^{2}}{\sin^{2}\theta_{A}}-\frac{L_{A}^{2}}{\tan\theta_{A}}\\ L_{B}=L_{A}+2h\tan\frac{\theta_{T}}{2}-h_{\mathrm{imm}}\tan\frac{\theta_{T}}{2} \end{array}$$

When the droplet expands from point B to point C, we obtain:

(3) $$\{\begin{array}{l} \frac{\theta_{C}L_{C}^{2}}{\sin^{2}\theta_{C}}-\frac{L_{C}^{2}}{\tan\theta_{C}}+h_{\mathrm{imm}}^{2}\tan\frac{\theta_{T}}{2}=\frac{\theta_{B}L_{B}^{2}}{\sin^{2}\theta_{B}}-\frac{L_{B}^{2}}{\tan\theta_{B}}\\ L_{C}=L_{B}+h_{\mathrm{imm}}\tan\frac{\theta_{T}}{2} \end{array}$$

When the droplet expands from point C to point D, we obtain:

(4) $$\{\begin{array}{l} \frac{\theta_{D}L_{D}^{2}}{\sin^{2}\theta_{D}}-\frac{L_{D}^{2}}{\tan\theta_{D}}=\frac{\theta_{C}L_{C}^{2}}{\sin^{2}\theta_{C}}-\frac{L_{C}^{2}}{\tan\theta_{C}}\\ L_{D}=L_{C}+p-2h\tan\frac{\theta_{T}}{2} \end{array}$$

In the composite wetting mode, when the three-phase contact point extends from point C to point D, the length of the interphase interface is the same as that of the full wetting mode. Due to the introduction of the infiltration depth *h*_imm_, the length of the interphase interface when the three-phase contact point extends from point A to point B and from point B to point C is slightly different from that of the full infiltration mode.

LG, SG, and SL, stand for liquid–gas interface, solid–gas interface, and solid–liquid interface, respectively.

**Figure 2:**
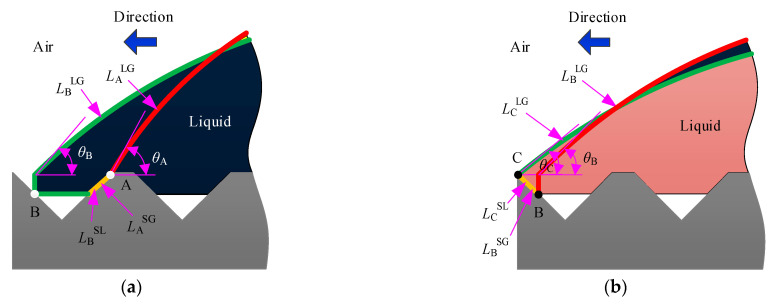
Schematic diagram of the interface changes between the phases of the composite infiltration mode: (**a**) A→B; (**b**) B→C.

As shown in Figure 2a, when the three-phase contact point extends from point A to point B, the length of each interface can be expressed by the following formula:

(5) $$\left\{ \begin{array}{l} \begin{array}{l} L_{A}^{\mathrm{LG}}=2\frac{\theta_{A}L_{A}}{\sin\theta_{A}}\\ L_{B}^{\mathrm{LG}}=2\frac{\theta_{B}L_{B}}{\sin\theta_{B}}+2h_{\mathrm{imm}}+4h\tan\frac{\theta_{T}}{2}-4h_{\mathrm{imm}}\tan\frac{\theta_{T}}{2} \end{array}\\ L_{A}^{\mathrm{SG}}=L_{B}^{\mathrm{SL}}=2\frac{h_{\mathrm{imm}}}{\cos\left(\theta_{T}/2\right)} \end{array}\right.$$

The free energy change $\Delta F_{A\to B}$ of the three-phase contact point extending from point A to point B can be deduced:

(6) $$\Delta F_{A\to B}=2\gamma^{\mathrm{LG}}\left(\frac{\theta_{B}L_{B}}{\sin\theta_{B}}-\frac{\theta_{A}L_{A}}{\sin\theta_{A}}+h_{\mathrm{imm}}+2h\tan\frac{\theta_{T}}{2}-2h_{\mathrm{imm}}\tan\frac{\theta_{T}}{2}\right)-2h_{\mathrm{imm}}\frac{\left(\gamma^{\mathrm{SG}}-\gamma^{\mathrm{SL}}\right)}{\cos\left(\theta_{T}/2\right)}$$

Dividing both sides by *γ*^LG^ at the same time, we obtain:

(7) $$\frac{\Delta F_{A\to B}}{\gamma^{\mathrm{LG}}}=2\left(\frac{\theta_{B}L_{B}}{\sin\theta_{B}}-\frac{\theta_{A}L_{A}}{\sin\theta_{A}}+h_{\mathrm{imm}}+2h\tan\frac{\theta_{T}}{2}-2h_{\mathrm{imm}}\tan\frac{\theta_{T}}{2}\right)-2h_{\mathrm{imm}}\frac{\cos\theta_{Y}}{\cos\left(\theta_{T}/2\right)}$$

Figure 2b shows the expansion of the three-phase contact point from point B to point C. The length of each interface is:

(8) $$\left\{ \begin{array}{l} \begin{array}{l} L_{B}^{\mathrm{LG}}=2\frac{\theta_{B}L_{B}}{\sin\theta_{B}}+2h_{\mathrm{imm}}\\ L_{C}^{\mathrm{LG}}=2\frac{\theta_{C}L_{C}}{\sin\theta_{C}} \end{array}\\ L_{B}^{\mathrm{SG}}=L_{C}^{\mathrm{SL}}=2\frac{h_{\mathrm{imm}}}{\cos\left(\theta_{T}/2\right)} \end{array}\right.$$

Similarly, the free energy change $\Delta F_{B\to C}$ of the three-phase contact point extending from point B to point C can be expressed as:

(9) $$\Delta F_{B\to C}=2\gamma^{\mathrm{LG}}\left(\frac{\theta_{C}L_{C}}{\sin\theta_{C}}-\frac{\theta_{B}L_{B}}{\sin\theta_{B}}-h_{\mathrm{imm}}\right)-2h_{\mathrm{imm}}\frac{\left(\gamma^{\mathrm{SG}}-\gamma^{\mathrm{SL}}\right)}{\cos\left(\theta_{T}/2\right)}$$

Dividing both sides by *γ*^LG^ at the same time, we obtain:

(10) $$\frac{\Delta F_{B\to C}}{\gamma^{\mathrm{LG}}}=2\left(\frac{\theta_{C}L_{C}}{\sin\theta_{C}}-\frac{\theta_{B}L_{B}}{\sin\theta_{B}}-h_{\mathrm{imm}}\right)-2h_{\mathrm{imm}}\frac{\cos\theta_{Y}}{\cos\left(\theta_{T}/2\right)}$$

The formula for the expansion of the three-phase contact point from point C to point D is consistent with the full infiltration model. Therefore, the two-dimensional mathematical model of the compound infiltration mode can be expressed by Equation (11):

(11) $$\left\{ \begin{array}{l} \begin{array}{l} L_{B}=L_{A}+2h\tan\frac{\theta_{T}}{2}-h_{\mathrm{imm}}\tan\frac{\theta_{T}}{2}\\ L_{C}=L_{B}+h_{\mathrm{imm}}\tan\frac{\theta_{T}}{2} \end{array}\\ L_{D}=L_{C}+p-2h\tan\frac{\theta_{T}}{2}\\ \frac{\theta_{B}L_{B}^{2}}{\sin^{2}\theta_{B}}-\frac{L_{B}^{2}}{\tan\theta_{B}}+4hh_{\mathrm{imm}}\tan\frac{\theta_{T}}{2}-3h_{\mathrm{imm}}^{2}\tan\frac{\theta_{T}}{2}=\frac{\theta_{A}L_{A}^{2}}{\sin^{2}\theta_{A}}-\frac{L_{A}^{2}}{\tan\theta_{A}}\\ \frac{\theta_{C}L_{C}^{2}}{\sin^{2}\theta_{C}}-\frac{L_{C}^{2}}{\tan\theta_{C}}+h_{\mathrm{imm}}^{2}\tan\frac{\theta_{T}}{2}=\frac{\theta_{B}L_{B}^{2}}{\sin^{2}\theta_{B}}-\frac{L_{B}^{2}}{\tan\theta_{B}}\\ \frac{\theta_{D}L_{D}^{2}}{\sin^{2}\theta_{D}}-\frac{L_{D}^{2}}{\tan\theta_{D}}=\frac{\theta_{C}L_{C}^{2}}{\sin^{2}\theta_{C}}-\frac{L_{C}^{2}}{\tan\theta_{C}}\\ \frac{\Delta F_{A\to B}}{\gamma^{\mathrm{LG}}}=2\left(\frac{\theta_{B}L_{B}}{\sin\theta_{B}}-\frac{\theta_{A}L_{A}}{\sin\theta_{A}}+h_{\mathrm{imm}}+2h\tan\frac{\theta_{T}}{2}-2h_{\mathrm{imm}}\tan\frac{\theta_{T}}{2}\right)-2h_{\mathrm{imm}}\frac{\cos\theta_{Y}}{\cos\left(\theta_{T}/2\right)}\\ \frac{\Delta F_{B\to C}}{\gamma^{\mathrm{LG}}}=2\left(\frac{\theta_{C}L_{C}}{\sin\theta_{C}}-\frac{\theta_{B}L_{B}}{\sin\theta_{B}}-h_{\mathrm{imm}}\right)-2h_{\mathrm{imm}}\frac{\cos\theta_{Y}}{\cos\left(\theta_{T}/2\right)}\\ \frac{\Delta F_{C\to D}}{\gamma^{\mathrm{LG}}}=2\left(\frac{\theta_{D}L_{D}}{\sin\theta_{D}}-\frac{\theta_{C}L_{C}}{\sin\theta_{C}}\right)-2\left(p-2h\tan\frac{\theta_{T}}{2}\right)\cos\theta_{Y} \end{array}\right.$$

Similarly, suppose stage A is the initial state, and the input conditions of the equation group include *θ*_A_, *θ*_T_, *θ*_Y_, *L*_A_, $\gamma^{\mathrm{LG}}$, *p*, *h*, and *h*_imm_. The output of the equation group is still *L*_B_, *L*_C_, *L*_D_, *θ*_B_, *θ*_C_, *θ*_D_, $\Delta F_{A\to B}$, $\Delta F_{B\to C}$, and $\Delta F_{C\to D}$. The compound infiltration model also contains nine equations and nine unknowns, and the model is solvable.

**Figure 3:**
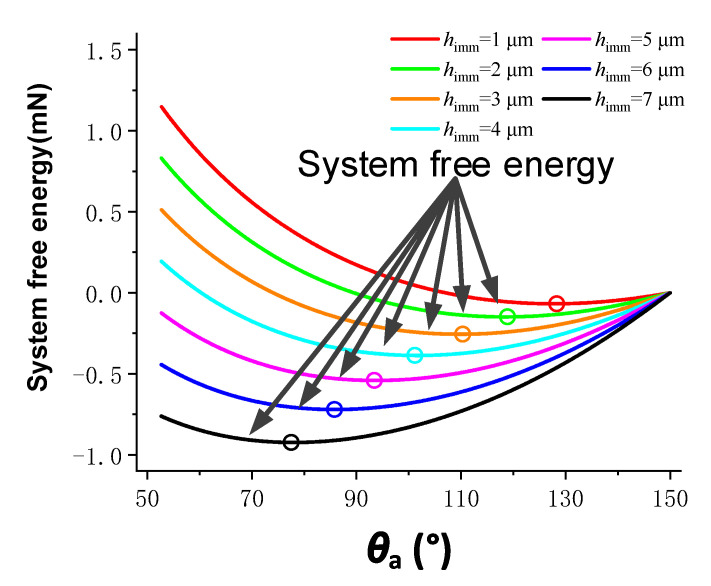
Calculation results of the composite infiltration model.

The relationship between system free energy and apparent contact angle in the compound infiltration mode was also obtained by iterative calculation of Equation (11) by constructing a loop program in MATLAB. The infiltration depth *h*_imm_ was increased from 1 μm to 7 μm in steps of 1 μm. The calculation results of the composite infiltration model are shown in Figure 3. When the infiltration depth was constant, the free energy of the system decreased first and then increased with the decrease of the apparent contact angle. Similarly, the apparent contact angle of the droplet when the surface of the microstructured array was stable could be obtained by determining the minimum value of the free energy.

**Figure 4:**
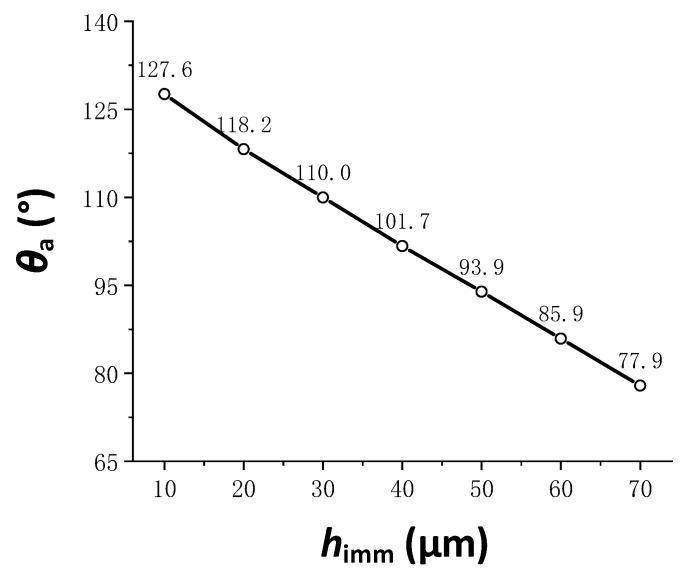
The relationship between the apparent contact angle and droplet infiltration depth.

The relationship between the steady state apparent contact angle and the droplet infiltration depth is shown in Figure 4, and the relationship between the two was basically linear. As the depth of infiltration increased, the apparent contact angle gradually decreased, because the droplet infiltration mode gradually approached the full infiltration mode.

In summary, comparing the calculation results of the simulation model with the calculation results of the mathematical model, it can be seen that the calculation results of the total infiltration model had a large error, and the calculation results of the composite infiltration model at *h*_imm_ = 3 μm were consistent with the simulation results. When the microstructure pitch *p* = 20 μm, the width *w* = 16 μm, the depth *h* = 8 μm, and the bottom angle *θ*_T_ = 90°, the microstructure array appears to be hydrophobic. Therefore, in this paper, the composite infiltration model was used as a theoretical model for the regulation of the wettability of the microstructure array surface.

The microstructure array processing tool is a diamond tip with a tip angle of 90°, then *θ*_T_ = 90°, and the relationship between the width of the processed microstructure and the depth of the microstructure is *w* = 2*h*. Therefore, different microstructure morphologies can be obtained by setting different *h* and *p*.

**Figure 5:**
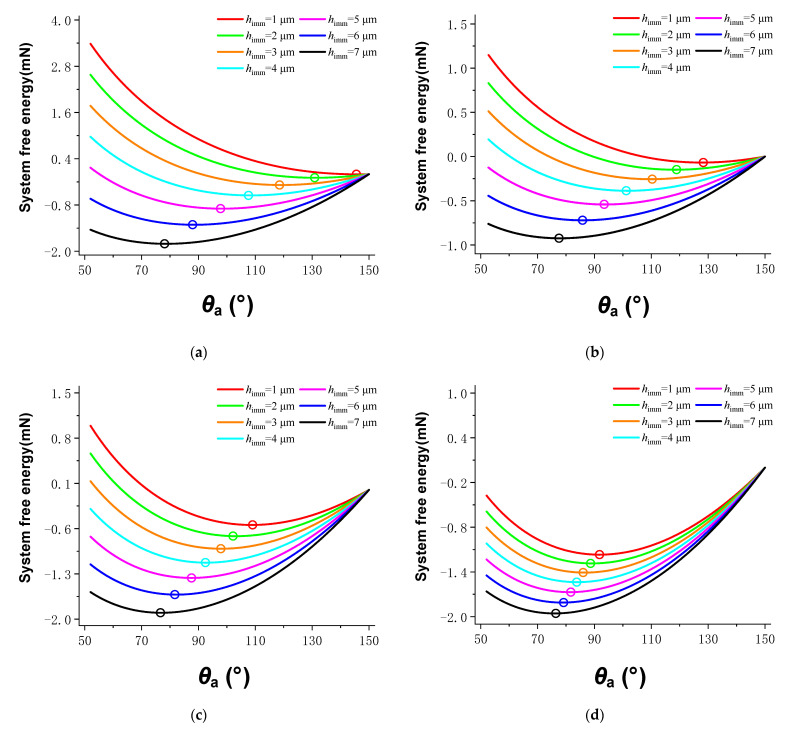
Apparent contact angle and system free energy under different microstructure spacings. (**a**) *p* = 16 μm; (**b**) *p* = 20 μm; (**c**) *p* = 30 μm; (**d**) *p* = 60 μm.

When the microstructure depth *h* is fixed, the influence of the microstructure pitch *p* on the wettability of the coating surface is studied. Using the composite infiltration model, we set *h* to 8 μm, and calculated the relationship between the free energy of the system and the apparent contact angle *θ*_a_ when the microstructure spacing *p* was 16, 20, 30, and 60 μm. The results are shown in Figure 5.

**Figure 6:**
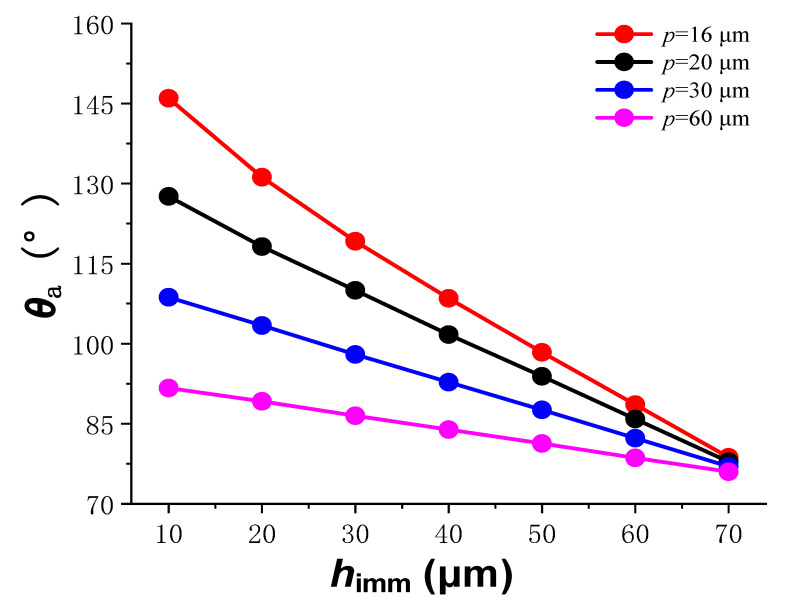
Variation range of apparent contact angle under different microstructure spacings.

The apparent contact angle corresponding to the minimum system free energy is the apparent contact angle when the liquid is stable. At different *p* values, there was a range of variation in *θ*_a_ with the infiltration depth *h*_imm_, and the results are shown in Figure 6. When *h* was fixed, with the increase of *p*, the variation range of *θ*_a_ gradually decreased, and the surface of the microstructure array gradually tended to be hydrophilic. This is because when *h* was fixed, *w* was also a fixed value, and an increase in *p* led to an increase in the contact area between the droplet and the surface of the Ni-P coating. According to the Cassie–Baxter model, the above situation increases the ratio *f*_a_ between the actual contact area of the liquid and the solid to the visible contact area, resulting in a decrease in the apparent contact angle. 

**Figure 7:**
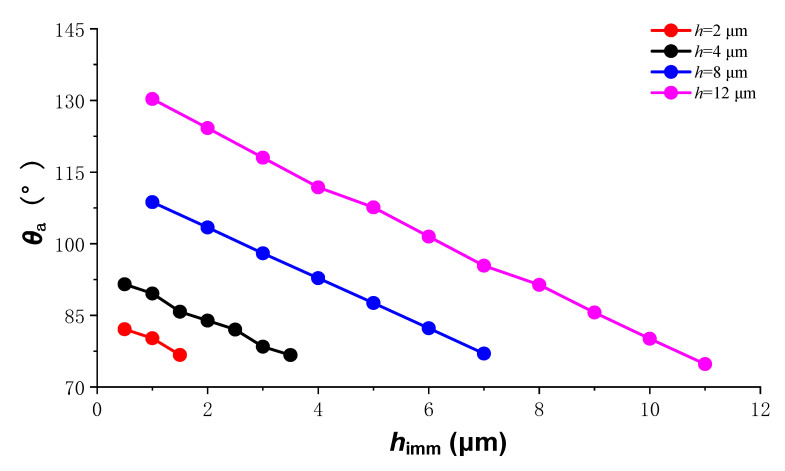
Variation range of apparent contact angle at different microstructure depths.

When the microstructure pitch *p* is fixed, the influence of the microstructure depth *h* on the wettability of the coating surface is studied. We set *p* to 30 μm, calculated the change of the stable apparent contact angle *θ*_a_ of the droplet when the microstructure depth *h* was 2, 4, 8 and 12 μm, and the results are shown in Figure 7. When *p* is a certain value, with the increase of *h*, the variation range of *θ*_a_ gradually increased, and the surface wettability of the microstructure array included both hydrophilic and hydrophobic ones.

Comparing the results in Figure 6 and Figure 7, the following correspondence can be found: the change curves of *p* = 20, 30, and 60 μm in Figure 6 correspond to the change curves of *h* = 12, 8, and 4 μm in Figure 7, respectively. In Figure 6, *h* is 8 μm, and *h*/*p* is 6/15, 4/15, and 2/15, respectively; in Figure 7, *p* is 30 μm, and *h*/*p* is also 6/15, 4/15, and 2/15 respectively. It can be seen that the change of the apparent contact angle is related to the ratio of the microstructure depth *h* to the microstructure pitch *p*. The surface wettability of the electroless Ni-P coating can be adjusted by processing microstructure arrays with different *h*/*p* values.

Considering the machinability of the large-area microstructure array on the surface of the electroless Ni-P coating, when *h* is small, the efficiency of large-area processing is too low; when *h* is large, large-area processing is likely to cause tool wear. Therefore, in the subsequent microstructure array processing experiments in this paper, *h* was set to 8 μm and *p* was set to 16, 20, 30, and 60 μm, respectively, and *h*/*p* was 7.5/15, 6/15, 4/ 15 and 2/15.

**Figure 8:**
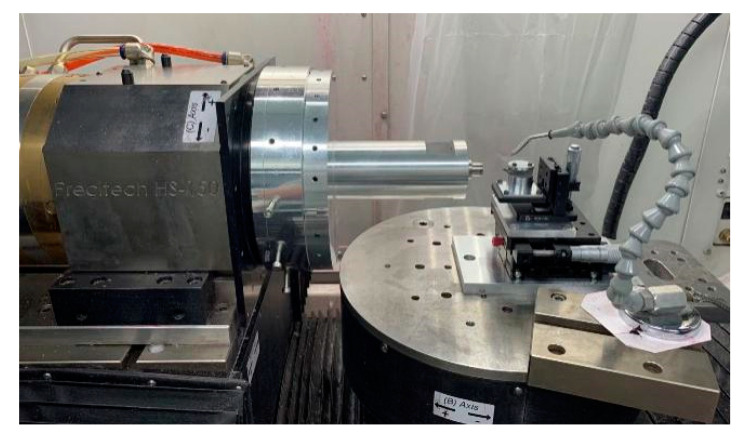
Ultra-precision machining machine tool.

The test was completed by the Nanoform X ultra-precision processing machine tool from Precitech, USA. The structure of the machine tool is shown in Figure 8. The inside of the machine tool is a T-shaped layout processing platform, which realizes processing through four-axis linkage, where the *X*-axis and *Z*-axis are moving axes, and the C axis and the B axis are the rotation axis. The maximum stroke of the moving axis is 220 mm, the maximum feed speed is 4000 mm/min, the programming resolution is 0.01 nm, the position feedback resolution is 0.016 nm, and the maximum speed of the C axis of the rotary axis is 1500 r/min; the maximum speed of working spindle can reach 10,000 r/min, the position feedback resolution is 0.025 arc second, the positioning accuracy is ±1 arc second, the rotary axis B axis position feedback resolution is 0.004 arc second, and the positioning accuracy is ±1 arc second. The performance parameters of the machine tool mentioned above all provide a strong guarantee for the development of cutting tests.

**Figure 9:**
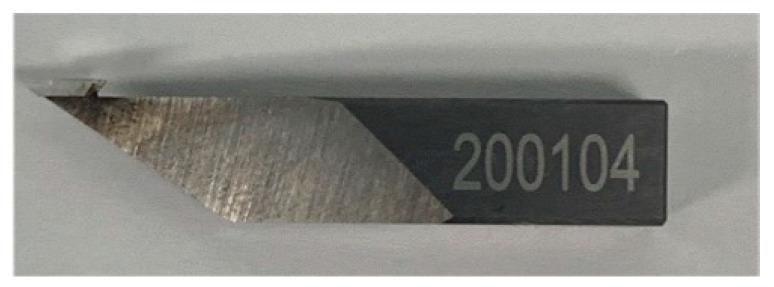
Diamond tool.

The cutting process uses a diamond cutting tool with a rake angle of 0° and a clearance angle of 15°, which can process microstructures of different sizes, as shown in Figure 9.

**Figure 10:**
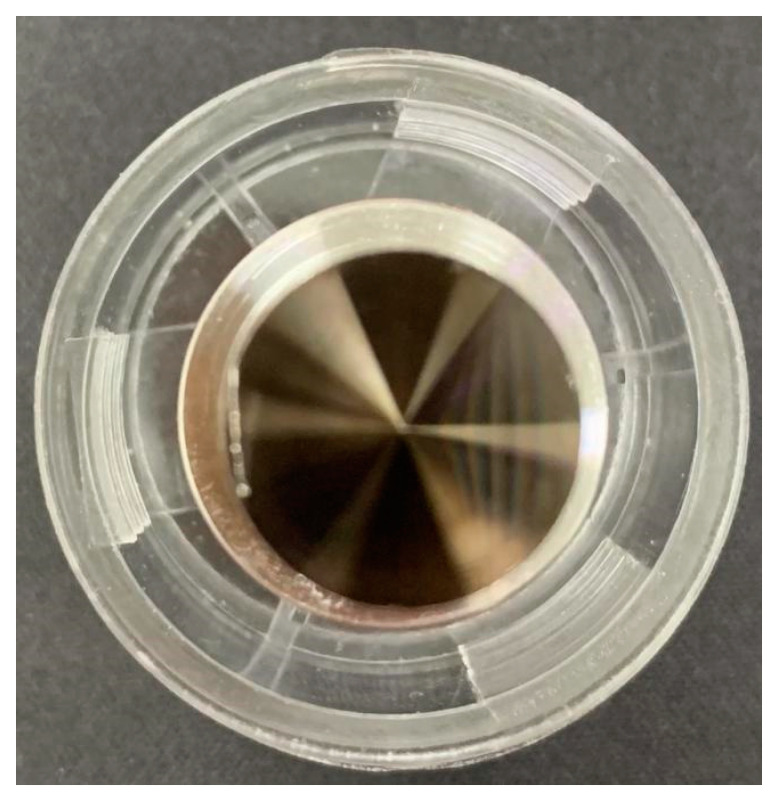
Cemented carbide mold.

In the experiment, ultra-precision machine tools were used to process different sizes of microstructures on Ni-P coating molds, as shown in Figure 10. By observing the inclined surface with microstructures of different widths under laser scanning confocal microscope (LEXT OLS5000, Olympus, Japan), the average surface roughness was 20 nm, which has high processing quality and facilitates the flow of PDMS during injection and the separation of PDMS during demolding [19].

**Figure 11:**
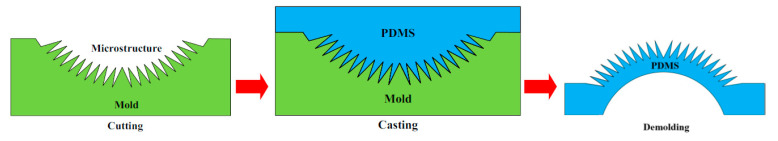
Sketch of the demolding process.

**Figure 12:**
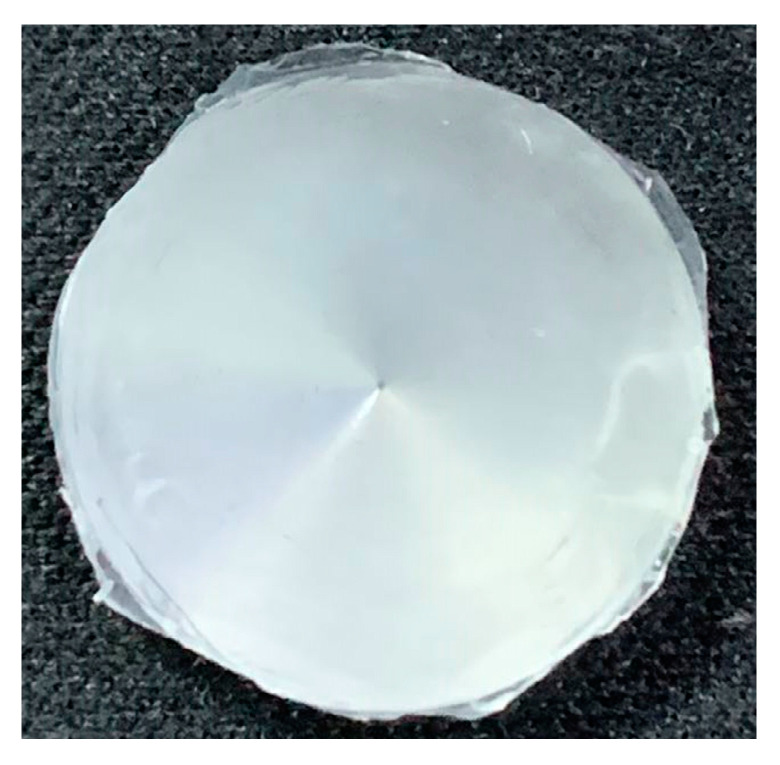
PDMS material with microstructure.

The PDMS material purchased from Dow Silicones Corporation, which has the advantages of low surface energy, chemical stability and low cost. In the experiment, firstly, we mixed the basic component (SYLGARD 184A) and the curing agent (SYLGARD 184B) at a mass ratio of 10:1, and then vacuumed until the bubbles inside were completely removed. Subsequently, the liquid PDMS was poured into the surface of the mold with wedged slanted microstructure, and a punch was used to press the PDMS, and cured at 80°C for 2 h. Finally, it was cooled down at room temperature, and the PDMS gradually removed from the mold from the edge to the middle. The demolding process is briefly shown in Figure 11, and the PDMS material with microstructure is shown in Figure 12.

**Figure 13:**
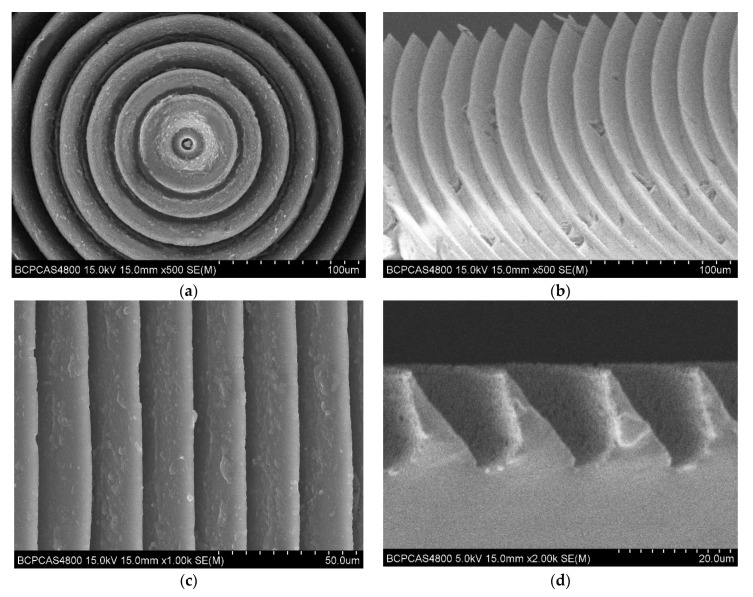
SEM image of PDMS material with microstructure. (**a**) Overall outline; (**b**) partial outline; (**c**) top view; (**d**) side view.

The measurement of the PDMS bionic structure was carried out on a HITACHI S-4800. The outline of the microstructure is shown in Figure 13. The overall outline of the PDMS bionic structure is shown in Figure 13a. It can be seen that a series of concentric circles were clearly visible, and the microstructure had a certain tilt angle, which is consistent with the designed microstructure of the mold. The specific tilt can be seen in Figure 13b. It can be observed in the partially enlarged microstructure, and rows of microstructures were arranged neatly and the structure was relatively complete. There will be a small amount of PDMS fragments remaining in the demolding process in the microstructure. The partial top view of the microstructure is shown in Figure 13c. The microstructure of each part had good parallelism and completeness. The side view of the microstructure is shown in Figure 13d. The overall microstructure had a certain slope and is triangular. The structural integrity was relatively complete, and the designed bionic microstructure was well realized.

## 3. Results

**Figure 14:**
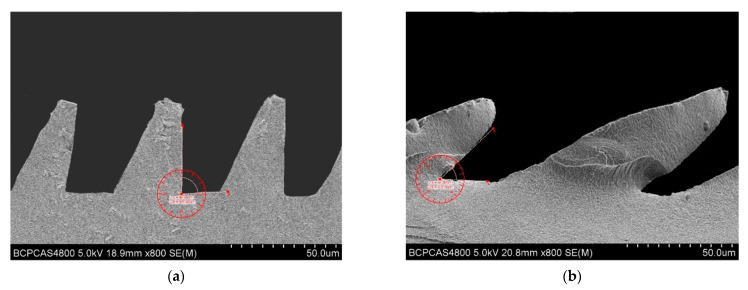
Bionic microstructures at different tilt angles. (**a**) Vertical microstructure; (**b**) 45° tilted microstructure.

In the experiment, microstructures with different inclination angles were designed. Among them, the vertical wedge-shaped structure and the 45° tilted microstructure are shown in Figure 14. Figure 14a is a vertical wedge structure with a width of 30 μm. There was no sharp corner at the top of the structure, because the size of the microstructure decreased. When the PDMS was poured into the microstructure of the cemented carbide mold, due to the viscosity and fluidity of the material of PDMS, a part of the air remained to be sealed at the bottom of the mold microstructure, which cannot be completely ruled out. When the microstructure was copied with PDMS, it appeared that the top of the microstructure was not a sharp triangle, but a flat surface. When the width of the microstructure was 60 μm, as shown in Figure 14b, the inclination angle of the bionic structure reached 45°, and the PDMS material could completely fill all the spaces of the microstructure of the cemented carbide mold, but due to the inclination of the microstructure, stress concentration resulted at the bottom during demolding, and prone to partial shedding, making the root of the PDMS material microstructure incomplete.

**Figure 15:**
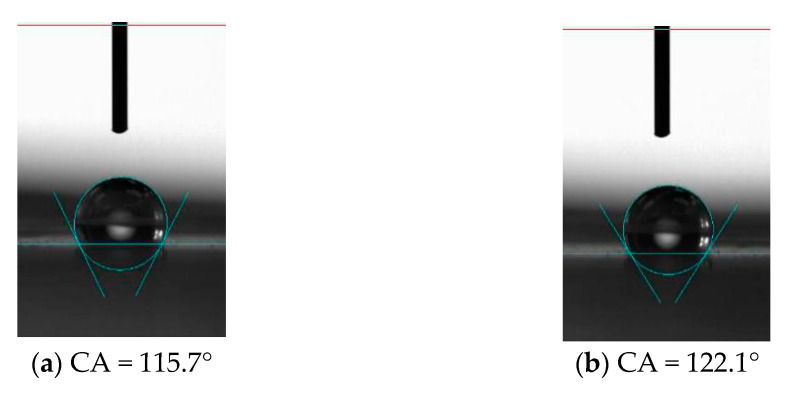
Non-microstructured PDMS planar hydrophobic properties. (**a**) Surface before processing; (**b**) surface after processing.

**Figure 16:**
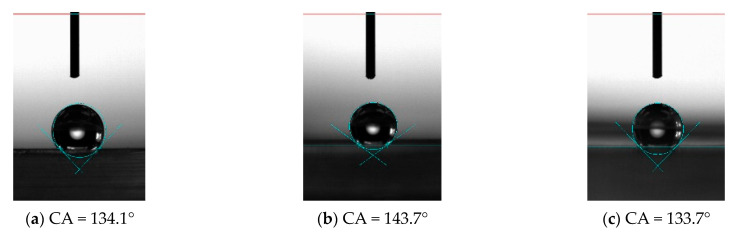
Hydrophobic characteristics of microstructures with different tilt angles when the microstructure width is 100 μm. (**a**) 90°; (**b**) 45°; (**c**) 30°.

**Figure 17:**
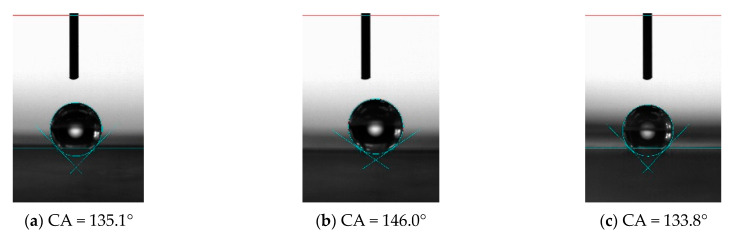
Hydrophobic characteristics of microstructures with different tilt angles when the microstructure width is 60 μm. (**a**) 90°; (**b**) 45°; (**c**) 30°.

**Figure 18:**
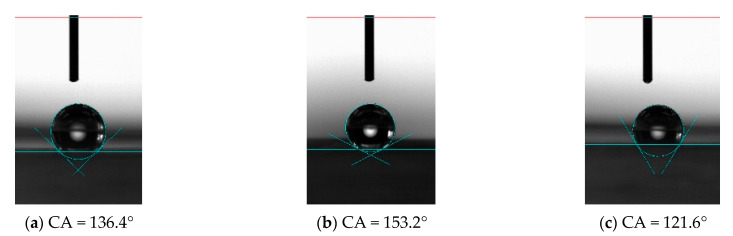
Hydrophobic characteristics of microstructures with different tilt angles when the microstructure width is 30 μm. (**a**) 90°; (**b**) 45°; (**c**) 30°.

For PDMS without microstructure, the contact angle of the surface was 115.7°, while after processing the microstructure, the contact angle of the unprocessed area on the PDMS surface was 122.1°, which increased slightly, indicating that the processed microstructure or the machining process has a certain influence on the properties of the material in the unprocessed area. It also shows that the hydrophobic properties of the material can be changed by processing the microstructure, as shown in Figure 15. The factors that affect the hydrophobic properties of materials include material properties and microstructure. Microstructure influences the hydrophobic properties of materials through its structural size, including length, width, height and tilt. For the vertical bionic structure, when the bionic structure decreased from 100 μm to 30 μm, the corresponding contact angles were 134.1° (Figure 16a), 135.1° (Figure 17a), and 136.4° (Figure 18a), respectively, and the contact angle tended to increase. For the inclined angle of 45°, the contact angle had the same trend. However, when the inclination angle was 30° and the microstructure width decreased from 60 μm to 30 μm, the contact angle decreased from 133.7° (Figure 17c) to 121.6° (Figure 18c). It is observed that when the microstructure width was 30 μm and the inclination angle was 30°, the microstructure integrity of the prepared PDMS was poor. The defect of the bottom of the microstructure can affect the functional characteristics of the microstructure and reduce the contact angle. Similarly, for the microstructures with the same width, when the width of the bionic structure was 60 μm, the contact angles of the microstructures changed to 135.1° (Figure 17a), 146.0° (Figure 17b), and 133.7° (Figure 17c) as the tilt angle decreased from 90° to 30°, and the contact angles first increased and then decreased. The same trend also occurred when the width was 100 μm and 30 μm, indicating that the contact angle of the microstructure increased with the decrease of the tilt angle, but when the tilt angle was small, it posed a higher challenge to the preparation process. The simulation results showed that the smaller the tilt angle is, the smaller the contact angle is. However, for the microstructure with a tilt angle of 30°, the root tear very easily occurred during the demolding, which rendered the microstructure no longer complete, thus affecting its functionality.

In the experiment, when the microstructure size was 30 μm and the inclination angle was 45°, as shown in Figure 18b, the contact angle of the microstructure was the largest, 153.2°, which is higher than the definition of the stable contact angle of the superhydrophobic material surface (>150°). By processing the microstructure, the contact angle of PDMS increased from 115.7° to 153.2°, and the hydrophobicity of the material changed from hydrophobicity to superhydrophobicity, indicating that it is an effective and efficient method to improve the hydrophobicity of the material by machining the microstructure of different sizes on the surface of the material.

## 4. Conclusions

In this paper, a two-dimensional mathematical model of the composite infiltration mode considering the hydrophobic properties of the microstructure parameters was established, the influence of the microstructure width and inclination angle on the hydrophobic properties of materials was discussed, and it was prepared by ultra-precision cutting and molding. The superhydrophobic structure (CA = 153.2°) was fabricated by the method, which improved the hydrophobic properties of the material.

## Data Availability

The data presented in this study are available from the corresponding authors upon reasonable request.
